# Associations between COVID-19 risk perceptions and smoking and quitting behavior among U.S. adults

**DOI:** 10.1016/j.abrep.2021.100394

**Published:** 2021-11-27

**Authors:** Amy L. Nyman, Claire A. Spears, Victoria Churchill, Vuong V. Do, Katherine C. Henderson, Zachary B. Massey, Reed M. Reynolds, Jidong Huang

**Affiliations:** a Georgia State University, School of Public Health, USA; b Department of Health Policy & Behavioral Sciences, School of Public Health, Georgia State University, USA; c Georgia State University, School of Public Health, USA; d Georgia State University, School of Public Health, USA; e Department of Health Policy & Behavioral Sciences, School of Public Health, Georgia State University, USA; f Strategic Communication, School of Journalism, University of Missouri, USA; g Georgia State University, School of Public Health, USA; h Department of Health Policy & Behavioral Sciences, School of Public Health, Georgia State University, USA

**Keywords:** Cigarette smoking, Cessation, COVID-19, Risk perceptions

## Abstract

**Objectives:**

The COVID-19 pandemic may impact cigarette smokers’ behaviors. Among smokers, perceptions about the risks of contracting COVID-19 and the severity of COVID-19 illness likely vary, and perceptions may be associated with individual smoking behavior. Our study measured smokers’ perceptions of COVID-19 risks and their association with smoking and quitting outcomes.

**Methods:**

A sample of 1,223 U.S. adult cigarette smokers participated in an online survey in October-November 2020 to assess their COVID-19-related risk perceptions and changes in smoking, readiness to quit, and quit attempts during the COVID-19 pandemic.

**Results:**

More smokers believed smoking could increase the severity of COVID-19 (43.6%, 95% CI: 40.1, 47.3) than believed smoking makes them more susceptible to COVID-19 (20.0%, 95% CI: 17.2, 23.0.). While there were no associations between perceptions of COVID-19 susceptibility and smoking behaviors or intentions, perceptions of greater smoking-related COVID-19 severity were associated with both higher likelihood of smoking increases (aOR: 2.16, 95% CI: 1.19, 3.93) and greater readiness to quit smoking (aOR: 1.65, 95% CI: 1.18, 2.30). Greater perceptions of general COVID-19 severity were associated with higher likelihood of smoking reductions (aOR: 1.12, 95% CI: 1.02, 1.22), greater readiness to quit smoking (aOR: 1.14, 95% CI: 1.07, 1.22), and higher likelihood of making a quit attempt (aOR: 1.12, 95% CI: 1.04, 1.22).

**Conclusions:**

Smokers’ perceptions about COVID-19 severity are related to their smoking behavior and likelihood of making a quit attempt. Providing smokers with accurate information on the relationship between smoking and the severity of COVID-19 may alter smoking behaviors.

## Highlights

• Smokers are more likely to think smoking increases risk of COVID-19 severity than susceptibility.
• Perceiving risk of COVID-19 severity is associated with readiness to quit and quit attempts.
• Perceiving risk of COVID-19 severity is also linked with both smoking increases and decreases.
• Greater psychological distress is associated with changes in smoking and quitting behavior.

## 1 Introduction

While cigarette smoking is well-documented as the leading cause of preventable death (Lariscy, 2019), scientists are now investigating the link between smoking and COVID-19 susceptibility and severity. Studies have found that smokers infected with COVID-19 may be more likely to become severely ill than those who do not smoke (Berlin et al., 2020, Heydari and Arfaeinia, 2021, Patanavanich and Glantz, 2020, Reddy et al., 2021, Zhang et al., 2021, Zhao et al., 2020) but it remains controversial whether smoking affects susceptibility to COVID-19 infection (Heydari and Arfaeinia, 2021, Prinelli et al., 2021, Simons et al., 2020, van Westen-Lagerweij et al., 2021). To promote public health, it is crucial to understand how COVID-19 impacts smokers and what factors may increase or decrease smoking behavior during the pandemic.

Research on smoking behavior during the early months of the COVID-19 pandemic indicated that smokers responded with both positive and negative health behaviors. For example, some smokers reported increased interest in quitting (Pettigrew et al., 2020), increased quit attempts (Jackson et al., 2020), reductions in smoking (Pišot et al., 2020), or increases in cessation (Jackson et al., 2020, Kayhan Tetik et al., 2020, Theuerkauff and Hanak, 2020). In contrast, other studies reported increases in smoking rates (Matsungo and Chopera, 2020, Mistry et al., 2021, Yan et al., 2020) and low numbers of quit attempts among smokers (Tattan-Birch et al., 2020). Still others found a combination of smoking increases and decreases (Bommelé et al., 2020, Grogan et al., 2020, Siddiqi et al., 2020, White et al., 2021, Yingst et al., 2021), or increased interest in quitting coupled with increased smoking rates (Streck et al., 2020). Pandemic-induced stress and worry were also found to be associated with both increases and decreases in smoking (Bommelé et al., 2020, Carreras et al., 2021, Patwardhan and Driscoll, 2020), inhibited quitting behavior (Joyce et al., 2021), and coping motives for smoking (Shepherd et al., 2021).

Smokers’ behavior in response to the COVID-19 pandemic may be related to their perceptions of illness risk. Both previous research and the Health Belief Model (Jones et al., 2015) suggest that risk perceptions are correlated with health-related behaviors (Brewer et al., 2004, Jones et al., 2015), including decisions about tobacco use (Nyman et al., 2019, Popova et al., 2018). In a survey conducted at the beginning of the COVID-19 pandemic, U.S. adults who perceived greater consequences from COVID-19 were more likely to adopt protective behaviors such as frequent handwashing, avoiding crowds, and social distancing (Bruine de Bruin & Bennett, 2020). Similarly, COVID-19 risk perceptions may influence smoking behaviors and quit attempts. Smokers who perceive their smoking habit to present a greater COVID-19 risk may attempt to quit or cut down on the amount they smoke or otherwise alter their smoking habits.

Studies of COVID-19 risk perceptions among smokers conducted within the early months of the pandemic found that perceived risk of severe COVID-19 illness was associated with increased interest in quitting smoking (Chertok, 2020, Elling et al., 2020, Gold et al., 2021, Klemperer et al., 2020, Streck et al., 2020) and greater perceived ease of quitting among treatment-seeking smokers (Rosoff-Verbit et al., 2021). Additionally, one study found a reduction in smoking among those perceiving a higher risk of COVID-19 infection (Gold et al., 2021, White et al., 2021). Only one study considered both types of risk perceptions concerning a single outcome measure (motivation to quit smoking) (Elling et al., 2020), while the rest considered either smoking-related COVID-19 risk perceptions or general COVID-19 risk perceptions, only. Examining several types of risk perceptions in relation to different smoking and quitting outcomes may provide a greater understanding of the relationship between COVID-19 risk perceptions and smokers’ behaviors.

Given rates of community spread, behavior, and knowledge about the transmission and consequences of COVID-19 have rapidly changed since the beginning of the pandemic, ongoing assessment of the population’s perceptions and responses is needed. Our study uses survey data from a nationally representative sample of U.S. smokers to understand relationships between several different risk perceptions and smoking and quitting behaviors more than six months after the beginning of the COVID-19 pandemic. This study aims to characterize the COVID-19 risk perceptions of adult cigarette smokers and to assess the degree to which COVID-19 risk perceptions are associated with changes in the amount of smoking, readiness to quit smoking, and quit attempts among U.S. adult cigarette smokers. As risk perception theory posits that many factors may influence an individual’s risk perceptions (Dryhurst et al., 2020), our study also accounts for demographic, psychological, and situational factors that may be associated with both perceptions and behavior. Specifically, our study aimed to answer the following questions: 1) What do smokers believe about their COVID-19 susceptibility and illness severity risks? 2) How are these risk perceptions related to changes in amount of smoking, readiness to quit smoking, and quit attempts made during the COVID-19 pandemic? 3) What other characteristics are associated with COVID-19 risk perceptions, smoking behavior, readiness to quit, and quit attempts?

## 2 Methods

### 2.1 Study sample and procedures

Data come from an October-November 2020 online survey of a national probability sample drawn from Ipsos Public Affairs’ KnowledgePanel, a probability-based web panel designed to be representative of non-institutionalized U.S. adults. Computers with internet access were provided to panelists who did not have them. KnowledgePanel members received small cash or prize incentives for their participation. Adult panelists (18 + years) who had reported current cigarette smoking or use of electronic nicotine delivery systems (ENDS) on recent Ipsos’ profile surveys were randomly sampled and invited to participate upon confirmation that they were currently or had recently (since February 2020) smoked cigarettes or used ENDS. Overall, 2,752 KnowledgePanel members were invited to participate in the survey, of which 1,630 (59.2%) completed the screener survey. Of the 1,535 qualified screener completers, nine were excluded for completing the survey in less than one-third of the median duration time (14.37 min), resulting in 1,526 cases, including our analytic sample of 1,223 current cigarette smokers. Participants who did not report currently smoking cigarettes (former smokers and non-smoking ENDS users) were not included in the present analysis. A final stage completion rate of 55.5% (number of final cases out of number invited) and a qualification rate of 94.2% (number of qualified participants out of number who completed the screener) were obtained. The average panel recruitment rate for this study, reported by Ipsos, was 11.3%, and the average profile rate was 62.4% for a cumulative response rate of 4.2% (Callegaro & DiSogra, 2009). A study-specific post-stratification weight was computed using an iterative proportional fitting (raking) procedure using benchmarks obtained from the 2019 National Health Interview Survey data (gender, race-ethnicity, census region, metropolitan status, education) and KnowledgePanel profile data (household income). The Georgia State University Institutional Review Board approved this study.

### 2.2 Measures

Complete wording and coding information on all measures can be found in Appendix A.

### 2.3 Dependent variables: Smoking and quitting behavior

To measure changes in the amount of smoking, smokers were asked how their cigarette use changed since February 2020 (the start of the COVID-19 pandemic) with response options ‘I increased my cigarette use,’ ‘My cigarette use has stayed the same,’ ‘I decreased my cigarette use,’ and ‘Other.’ The ‘other’ category contained minimal responses and was excluded from analyses. Readiness to quit was measured by the following, “Select the number that indicates where you are now in your thinking about quitting smoking,” with a 0–10 scale ranging from ‘No thought of quitting’ to ‘I am now taking action to quit.’ The midpoint of 5 was labeled, ‘Think I should quit but not quite ready.’ Following prior research on readiness to quit smoking using the contemplation ladder, which created groupings analogous to the first three stages of change (Prochaska & DiClemente, 1983), we collapsed this scale into three categories, representing the stages of change: precontemplation (0–2), contemplation (3–7), and preparation (8–10) (Herzog et al., 2000). To assess quit attempts, smokers were asked if they have made a serious attempt to quit smoking since February 2020.

### 2.4 Independent variables: COVID-19-related risk perceptions

As perceived severity and perceived susceptibility to risk have been established as components of the “perceived threat” construct in health and risk communication research (Popova, 2012), our study included both. COVID-19-related risk perceptions were assessed by four distinct questions, measuring the perceived risk of smoking-related COVID-19 susceptibility and smoking-related COVID-19 effects severity as well as the perceived risk of general COVID-19 susceptibility and general COVID-19 symptoms severity. To assess risk perceptions of smoking-related COVID-19 susceptibility and effects severity, we adapted items from Wave 4 of the Population Assessment of Tobacco and Health (PATH) study (Population Assessment of Tobacco and Health (PATH) Public-Use Files User Guide, 2021). Participants were asked how much they agree or disagree with the following statements: “Smoking cigarettes can cause me to be more likely to get coronavirus” and “Smoking cigarettes can cause me to have more severe effects of coronavirus.” Response options were ‘strongly disagree’, ‘disagree’, ‘neither disagree nor agree’, ‘agree’, ‘strongly agree’, and ‘don’t know’. The ‘don’t know’ responses represented fewer than 10% of cases and were excluded from analyses. To measure perceptions of general COVID-19 susceptibility and symptoms severity, participants were asked, “How likely do you think you are to be infected by the coronavirus over the next year?”: ‘unlikely’, ‘possible’, ‘likely’, ‘almost certain’, or ‘certain’, and “On a scale from 0 to 10, how severe do you think your symptoms will be if you become infected with coronavirus?” Only the anchors were labeled, with 0 labeled ‘I would likely have no symptoms’ and 10 labeled ‘I would likely die from it’.

### 2.5 Participant characteristics and other covariates

Adjustment variables included demographics (gender, age, race/ethnicity, education, and household income), and other characteristics possibly associated with smoking outcomes: ENDS use status, psychological distress (a sum of the Kessler 6 items (Kessler et al., 2002), self-efficacy for quitting (Jamieson & Jamieson, 2001), and nicotine dependence (Shadel et al., 2014) (a sum of responses to four variables measuring nicotine dependence). Changes in experiences since the beginning of the pandemic, such as cigarette accessibility and affordability, and amount of time spent in places allowing smoking, were also included.

### 2.6 Statistical analysis

Weighted percentages, means, and 95% Confidence Intervals (CI) were used to characterize risk perceptions and the sample overall. Weighted multinomial logistic regression was used to predict increases and decreases in smoking behavior, using “stayed the same” as the reference category. Weighted ordinal logistic regression was used to predict greater readiness to quit smoking, following a non-significant likelihood ratio test of the proportional odds assumption for the recoded three-level readiness to quit scale. Weighted binary logistic regression was used to predict a smoking quit attempt, using “no quit attempt” as the reference category. Each regression model predicting perceived COVID-19 risks incorporated both susceptibility and illness severity measures and adjusted for gender, age, race/ethnicity, education level, household income, ENDS use status, psychological distress, self-efficacy for quitting, nicotine dependence, cigarette accessibility, cigarette affordability, and time spent in places allowing smoking. A p-value less than 0.05 was considered statistically significant for all the tests. All analyses were conducted with Stata, version 15 (StataCorp LLC).

## 3 Results

### 3.1 Sample characteristics and smoking outcomes

Sample characteristics are detailed in Appendix B. Just under half the sample were female (46.6%), slightly more than two-thirds (69.4%) were non-Hispanic white, and nearly half (47.1%) reported an annual household income of between $30,000 and $99,999. Fewer than one in five respondents were current ENDS users (15.2%) or had recently quit using ENDS (2.7%). While most smokers did not change their amount of smoking during the pandemic (68.9%, 95% CI: 65.6, 72.1), roughly equal percentages either increased (16.1%, 95% CI: 13.7, 19.0) or decreased (14.9%, 95% CI: 12.6, 17.6) their amount of smoking. At the time of the survey, more than half of smokers were in the contemplation phase of readiness to quit smoking (61.5%, 95% CI: 58.0, 64.9), one-quarter were in the precontemplation phase (26.4%, 95% CI: 23.4, 29.6), and one in ten were preparing to quit (12.1%, 95% CI: 10.0, 14.6). Roughly one in five smokers (20.6%, 95% CI: 17.9, 23.6) reported making a quit attempt during the pandemic.

### 3.2 Risk perceptions

Only one in five smokers (20.0%, 95% CI: 17.2, 23.0) believed that smoking made them more susceptible to contracting COVID-19. Smokers ages 30–44 and those with college degrees were more likely than those of other ages or less educated, respectively, to believe that smoking could cause greater susceptibility to COVID-19. Close to half of smokers (43.6%, 95% CI: 40.1, 47.3) believed that smoking could cause them greater severity of COVID-19 if infected (Table 1). Older smokers and those identifying as Hispanic perceived a greater general likelihood of severe COVID-19 infection than younger smokers and those of other ethnicities, respectively.

**Table 1:** Smokers’ COVID-related risk perceptions by sociodemographic characteristics

		Smoking-related COVID-19 susceptibility and severity^a^				General COVID-19 Susceptibility and severity	
		*“Smoking cigarettes can* *cause me to be more likely* *to get coronavirus.”*		*“Smoking cigarettes can* *cause me to have more* *severe effects of coronavirus.”*		Perceived likelihood of COVID-19 infection^b^	Perceived severity of COVID-19 symptoms^c^
		Weighted % (95% C.I.) n = 1,142		Weighted % (95% C.I.) n = 1,146		Weighted mean (95% C.I.) n = 1,215	Weighted mean (95% C.I.) n = 1,214
		Disagree	Agree	Disagree	Agree		
	n^d^	n = 433	n = 216	n = 200	n = 515		
**Overall**	1,223	37.3 (33.9, 40.9)	20.0 (17.2, 23.0)	18.6 (15.9, 21.7)	43.6 (40.1, 47.3)	1.95 (1.89, 2.01)	5.15 (4.97, 5.32)
**Gender**: Male Female	678 545	36.3 (31.6, 41.1) 38.5 (33.4, 43.9)	20.2 (16.4, 24.6) 19.7 (16.0, 24.1)	17.7 (14.3, 21.6) 19.8 (15.6, 24.8)	47.5 (42.5, 52.6) 39.1 (34.3, 44.1)	1.90 (1.83, 1.98) 2.00 (1.90, 2.10)	5.01 (4.78, 5.23) 5.31 (5.03, 5.58)
**Age**: 18–29 30–44 45–59 60+	73 293 390 467	33.5 (22.1, 47.2) 35.2 (28.8, 42.3) 38.2 (32.9, 43.7) 41.4 (36.2, 46.9)	18.3 (10.6, 29.6) 29.0 (23.2, 35.6) 19.1 (15.0, 24.0) 10.9 (8.2, 14.5)	15.1 (7.8, 27.2) 18.0 (12.9, 24.5) 21.3 (17.0, 26.3) 18.4 (14.3, 23.3)	42.0 (29.7, 55.4) 50.2 (43.5, 56.9) 41.2 (35.8, 46.8) 39.4 (34.4, 44.8)	2.04 (1.85, 2.23) 2.07 (1.92, 2.22) 1.86 (1.78, 1.94) 1.84 (1.78, 1.90)	4.43 (3.92, 4.94) 5.08 (4.72, 5.43) 5.24 (4.95, 5.53) 5.62 (5.35, 5.90)
**Race/ethnicity**: White, NH Black, NH Other, NH Hispanic	920 138 65 100	38.2 (34.2, 42.3) 34.4 (25.1, 45.1) 36.5 (21.9, 54.1) 35.7 (24.5, 48.7)	17.9 (15.1, 21.2) 19.9 (12.7, 29.8) 25.4 (14.2, 41.2) 30.2 (19.7, 43.4)	17.3 (14.4, 20.7) 21.4 (13.7, 31.8) 20.2 (9.3, 38.4) 23.0 (13.9, 35.6)	44.8 (40.7, 49.0) 38.9 (29.3, 49.3) 35.9 (22.5, 51.9) 46.6 (34.7, 59.0)	1.96 (1.90, 2.02) 1.85 (1.69, 2.00) 1.84 (1.40, 2.29) 2.09 (1.80, 2.39)	5.11 (4.91, 5.31) 5.00 (4.47, 5.53) 4.87 (4.10, 5.65) 5.73 (5.10, 6.37)
**Education**: < High School High School Some College College grad+	111 424 476 212	48.4 (37.8, 59.1) 33.8 (28.5, 39.7) 38.4 (33.2, 43.9) 28.7 (21.7, 36.9)	13.7 (8.1, 22.3) 18.7 (14.4, 23.9) 20.2 (16.0, 25.2) 32.4 (24.3, 41.7)	29.3 (20.2, 40.4) 16.3 (12.8, 20.6) 17.1 (13.1, 21.9) 14.5 (9.2, 22.0)	31.6 (22.7, 42.1) 43.7 (37.8, 49.8) 46.4 (40.9, 52.0) 53.6 (44.4, 62.6)	1.99 (1.75, 2.22) 1.89 (1.80, 1.97) 1.97 (1.87, 2.06) 2.06 (1.94, 2.18)	5.26 (4.68, 5.83) 5.24 (4.97, 5.51) 5.05 (4.78, 5.33) 4.92 (4.53, 5.31)
**HH Income**: < $30,000 $30 K - $99,999 ≥ $100,000	374 619 230	39.3 (32.7, 46.3) 35.8 (31.3, 40.6) 37.9 (30.2, 46.1)	17.7 (13.0, 23.6) 19.7 (16.1, 23.8) 23.4 (17.1, 31.1)	20.1 (15.0, 26.4) 17.2 (13.8, 21.2) 19.8 (13.7, 27.5)	36.4 (30.4, 42.9) 45.9 (40.9, 50.8) 48.4 (40.1, 56.7)	1.98 (1.84, 2.12) 1.91 (1.85, 1.98) 1.99 (1.84, 2.14)	5.36 (5.02, 5.71) 5.15 (4.91, 5.39) 4.86 (4.47, 5.25)

Disagree includes both “disagree” and “strongly disagree;, Agree includes both “agree” and “strongly agree;, NH = Non-Hispanic; HH = Household.

^a^ Neutral percentages for these variables are not displayed in the table; Percentages neutral, disagree, and agree add to approximately 100%.

^b^ Scale 1–5, Higher number means greater perceived likelihood;

^c^ Scale 0–10, Higher number means greater perceived severity.

^d^n provided for all smokers.

### 3.3 Changes in smoking behavior

Controlling for beliefs about risk of illness severity, beliefs about participants’ risk of susceptibility to COVID-19 were not associated with changes in their cigarette smoking during the pandemic. However, controlling for beliefs about risk of illness susceptibility, smokers who believed that smoking could cause greater severity of COVID-19 effects were more likely to have increased their amount of cigarette smoking during the pandemic (aOR: 2.16, 95% CI: 1.19, 3.93) (Table 2). Those who believed they were generally at greater risk of severity for COVID-19 infection were more likely to have decreased their smoking during the pandemic (aOR: 1.12, 95% CI: 1.02, 1.22.) Being a current ENDS user, having lower nicotine dependence, and spending less time where smoking is allowed were all associated with decreases in smoking. Greater psychological distress and less cigarette affordability were associated with both increases and decreases in smoking (Table 2).

**Table 2:** Predictors of changes in cigarette smoking during the COVID-19 pandemic using Multinomial Logistic Regression

	Smoking-related COVID risks – aOR n = 1,086		General COVID risks – aOR n = 1,170	
	Increase	Decrease	Increase	Decrease
***“Smoking cigarettes can cause me to be more likely to get coronavirus.”*** Strongly disagree/disagree Neutral Strongly agree/agree	0.82 (0.42, 1.62) REF 0.87 (0.44, 1.69)	1.14 (0.64, 2.04) REF 1.50 (0.70, 3.19)		
***“Smoking cigarettes can cause me to have more severe effects of coronavirus.”*** Strongly disagree/disagree Neutral Strongly agree/agree	1.22 (0.47, 3.15) REF 2.16 (1.19, 3.93)**	1.31 (0.64, 2.67) REF 1.32 (0.67, 2.59)		
**Perceived likelihood of COVID infection**			0.98 (0.75, 1.29)	0.98 (0.74, 1.30)
**Perceived severity of COVID symptoms**			1.04 (0.94, 1.14)	1.12 (1.02, 1.22)*
**Gender** Male Female	REF 1.33 (0.83, 2.12)	REF 0.82 (0.53, 1.26)	REF 1.07 (0.68, 1.68)	REF 0.76 (0.50, 1.14)
**Age** 18–29 30–44 45–59 60+	REF 1.00 (0.38, 2.61) 0.96 (0.37, 2.51) 0.67 (0.25, 1.81)	REF 0.99 (0.44, 2.24) 0.97 (0.44, 2.14) 1.55 (0.70, 3.40)	REF 0.83 (0.35, 1.96) 0.72 (0.31, 1.71) 0.52 (0.21, 1.28)	REF 0.76 (0.35, 1.65) 0.69 (0.32, 1.51) 0.90 (0.41, 1.98)
**Race/ethnicity** White, NH Black, NH Other, NH Hispanic	REF 0.82 (0.39, 1.71) 0.93 (0.28, 3.13) 1.13 (0.50, 2.55)	REF 1.01 (0.57, 1.80) 0.86 (0.36, 2.05) 1.30 (0.57, 2.94)	REF 0.90 (0.46, 1.79) 0.86 (0.30, 2.51) 0.87 (0.41, 1.85)	REF 0.95 (0.53, 1.69) 1.09 (0.49, 2.45) 1.49 (0.71, 3.15)
**Education** < High School High School Some College College grad+	REF 0.56 (0.25, 1.23) 0.86 (0.39, 1.88) 1.09 (0.42, 2.86)	REF 1.16 (0.54, 2.50) 1.66 (0.79, 3.48) 1.69 (0.63, 4.53)	REF 0.75 (0.36, 1.55) 1.18 (0.56, 2.48) 1.73 (0.74, 4.07)	REF 1.37 (0.65, 2.92) 2.06 (1.00, 4.24)* 2.05 (0.80, 5.28)
**Household Income** < $30,000 $30 K - $99,999 ≥ $100,000	REF 0.79 (0.46, 1.37) 0.89 (0.45, 1.75)	REF 0.70 (0.42, 1.16) 0.69 (0.33, 1.44)	REF 1.00 (0.59, 1.69) 0.83 (0.43, 1.61)	REF 0.76 (0.46, 1.25) 0.82 (0.42, 1.60)
**ENDS use status** Current user Recent quitter Never use/long-term quitter	REF 2.02 (0.54, 7.59) 0.92 (0.41, 2.05)	REF 0.18 (0.03, 1.03) 0.47 (0.25, 0.88)*	REF 1.87 (0.57, 6.07) 0.86 (0.43, 1.73)	REF 0.15 (0.03, 0.83)* 0.41 (0.23, 0.75)**
**Psychological distress**	1.09 (1.04, 1.13)***	1.08 (1.03, 1.12)***	1.08 (1.04, 1.12)***	1.04 (1.00, 1.09)
**Lower self-efficacy for quitting**	1.24 (0.89, 1.75)	0.86 (0.64, 1.16)	1.27 (0.94, 1.73)	0.87 (0.65, 1.16)
**Nicotine dependence**	1.05 (0.97, 1.13)	0.92 (0.86, 0.97)**	1.04 (0.97, 1.12)	0.92 (0.87, 0.97)**
**Cigarettes harder to obtain**	1.20 (0.67, 2.16)	0.71 (0.35, 1.44)	1.31 (0.71, 2.42)	0.77 (0.41, 1.45)
**Cigarettes less affordable**	1.62 (1.05, 2.50)*	1.62 (1.04, 2.52)*	1.51 (1.00, 2.27)*	1.52 (0.99, 2.35)
**Less time spent where smoking allowed**	0.36 (0.21, 0.61)***	3.63 (2.22, 5.94)***	0.36 (0.21, 0.61)***	3.16 (1.96, 5.10)***

* p ≤ 0.05, ** p ≤ 0.01, *** p ≤ 0.001, “Stayed the same” is the reference category for changes in cigarette smoking.

### 3.4 Readiness to quit

Though risk perceptions of COVID-19 susceptibility were not associated with readiness to quit, those who believed smoking causes greater severity of infection (aOR: 1.65, 95% CI: 1.18, 2.30) and those who perceived greater general risk of severe COVID-19 infection (aOR: 1.14, 95% CI: 1.07, 1.22) expressed greater readiness to quit smoking (Table 3). In addition, older smokers, Black, non-Hispanic smokers, those with household incomes of $100,000 or more, and those with higher quitting self-efficacy and psychological distress expressed greater readiness to quit smoking.

**Table 3:** Predictors of readiness to quit smoking during the COVID-19 pandemic using Ordinal Logistic Regression

	Smoking-related COVID risks – aOR n = 1,092	General COVID risks – aOR n = 1,178
***“Smoking cigarettes can cause me to be more likely to get coronavirus.”*** Strongly disagree/disagree Neutral Strongly agree/agree	0.83 (0.57, 1.21) REF 1.06 (0.68, 1.65)	
***“Smoking cigarettes can cause me to have more severe effects of coronavirus.”*** Strongly disagree/disagree Neutral Strongly agree/agree	0.87 (0.52, 1.45) REF 1.65 (1.18, 2.30)**	
**Perceived likelihood of COVID infection**		1.03 (0.81, 1.31)
**Perceived severity of COVID symptoms**		1.14 (1.07, 1.22)***
**Gender** Male Female	REF 1.21 (0.89, 1.65)	REF 1.06 (0.79, 1.43)
**Age** 18–29 30–44 45–59 60+	REF 1.63 (0.86, 3.09) 2.28 (1.20, 4.32)** 2.50 (1.29, 4.85)**	REF 1.57 (0.85, 2.87) 1.89 (1.01, 3.50)* 1.80 (0.85, 3.39)
**Race/ethnicity** White, NH Black, NH Other, NH Hispanic	REF 1.74 (0.99, 3.08) 0.91 (0.48, 1.72) 0.98 (0.54, 1.77)	REF 1.80 (1.05, 3.10)* 0.90 (0.49, 1.63) 0.98 (0.58, 1.65)
**Education** < High School High School Some College College grad+	REF 0.95 (0.58, 1.55) 1.00 (0.61, 1.63) 0.89 (0.47, 1.68)	REF 1.08 (0.69, 1.69) 1.14 (0.73, 1.80) 0.97 (0.54, 1.75)
**Household Income** < $30,000 $30 K - $99,999 ≥ $100,000	REF 1.09 (0.74, 1.59) 1.84 (1.04, 3.25)*	REF 1.26 (0.89, 1.80) 2.24 (1.33, 3.75)**
**ENDS use status** Current user Recent quitter Never use/long-term quitter	REF 1.51 (0.68, 3.35) 0.79 (0.49, 1.28)	REF 1.40 (0.61, 3.23) 0.77 (0.49, 1.22)
**Psychological distress**	1.04 (1.01, 1.08)**	1.03 (1.00, 1.06)*
**Lower self-efficacy for quitting**	0.85 (0.67, 1.07)	0.79 (0.63, 1.00)*
**Nicotine dependence**	0.99 (0.94, 1.04)	0.98 (0.93, 1.03)
**Cigarettes harder to obtain**	0.64 (0.38, 1.07)	0.70 (0.45, 1.08)
**Cigarettes less affordable**	1.18 (0.86, 1.63)	1.24 (0.92, 1.67)
**Less time spent where smoking allowed**	1.28 (0.95, 1.71)	1.22 (0.93, 1.61)

* p ≤ 0.05, ** p ≤ 0.01, *** p ≤ 0.001.

### 3.5 Quit attempts

Beliefs about the risk of COVID-19 susceptibility were not associated with making a quit attempt. However, those who believed they were at greater general risk of severe COVID-19 infection were more likely to report making a quit attempt during the pandemic (aOR: 1.12, 95% CI: 1.04, 1.22) than those perceiving lower general risk of severe infection (Table 4). In general, female smokers, those ages 60 and over, and Black, non-Hispanic smokers were more likely to have made a quit attempt than male smokers, those ages 18–29, and White, non-Hispanic smokers, respectively. Greater psychological distress, greater self-efficacy for quitting, and greater cigarette accessibility were also associated with higher likelihood of quit attempts.

**Table 4:** Predictors of quit attempts during the COVID-19 pandemic using Binary Logistic Regression

	Smoking-related COVID risks – aOR n = 1,088	General COVID risks – aOR n = 1,174
***“Smoking cigarettes can cause me to be more likely to get coronavirus.”*** Strongly disagree/disagree Neutral Strongly agree/agree	0.95 (0.61, 1.50) REF 1.14 (0.66, 1.97)	
***“Smoking cigarettes can cause me to have more severe effects of coronavirus.”*** Strongly disagree/disagree Neutral Strongly agree/agree	0.74 (0.41, 1.35) REF 1.16 (0.72, 1.86)	
**Perceived likelihood of COVID infection**		0.90 (0.70, 1.16)
**Perceived severity of COVID symptoms**		1.12 (1.04, 1.22)**
**Gender** Male Female	REF 1.64 (1.12, 2.41)*	REF 1.37 (0.96, 1.96
**Age** 18–29 30–44 45–59 60+	REF 1.15 (0.50, 2.64) 1.59 (0.70, 3.61) 2.49 (1.07, 5.82)*	REF 0.92 (0.42, 2.02) 1.06 (0.48, 2.32) 1.38 (0.62, 3.08)
**Race/ethnicity** White, NH Black, NH Other, NH Hispanic	REF 2.63 (1.52, 4.54)*** 1.07 (0.47, 2.40) 1.66 (0.87, 3.18)	REF 2.25 (1.34, 3.76)** 0.95 (0.43, 2.09 1.83 (0.97, 3.43)
**Education** < High School High School Some College College grad+	REF 1.16 (0.62, 2.15) 0.95 (0.51, 1.79) 1.09 (0.49, 2.44)	REF 1.36 (0.74, 2.48) 1.36 (0.75, 2.48) 1.37 (0.63, 2.95)
**Household Income** < $30,000 $30 K - $99,999 ≥ $100,000	REF 0.91 (0.57, 1.48) 0.85 (0.45, 1.61)	REF 0.96 (0.62, 1.49) 0.95 (0.52, 1.73)
**ENDS use status** Current user Recent quitter Never use/long-term quitter	REF 1.32 (0.44, 3.91) 0.86 (0.48, 1.54)	REF 1.46 (0.51, 4.13) 0.85 (0.48, 1.51)
**Psychological distress**	1.06 (1.01, 1.10)**	1.04 (0.99, 1.08)
**Lower self-efficacy for quitting**	0.75 (0.58, 0.96)*	0.76 (0.60, 0.96)*
**Nicotine dependence**	1.00 (0.95, 1.06)	0.99 (0.94, 1.05)
**Cigarettes harder to obtain**	0.53 (0.29, 0.97)*	0.57 (0.32, 1.01)*
**Cigarettes less affordable**	1.16 (0.77, 1.75)	1.30 (0.90, 1.89)
**Less time spent where smoking allowed**	1.39 (0.97, 2.00)	1.32 (0.93, 1.88)

* p ≤ 0.05, ** p ≤ 0.01, *** p ≤ 0.001.

## 4 Discussion

Our findings suggest that six months into the pandemic, some COVID-19 risk perceptions continue to be associated with smoking and quitting behavior. The belief that smoking can cause greater severity of COVID-19 effects for smokers was associated with increases in smoking during the pandemic, while the perception of greater risk of severe symptoms if infected was associated with decreases in smoking during the pandemic. In addition, both smoking-related and general risk perceptions of COVID-19 illness severity were associated with greater readiness to quit. Finally, greater general perceived risk of COVID-19 symptoms severity was associated with making a quit attempt. These findings corroborate previous studies linking COVID-19 risk perceptions with smoking and quitting intentions and outcomes (Chertok, 2020, Elling et al., 2020, Gold et al., 2021, Klemperer et al., 2020, Rosoff-Verbit et al., 2021, Streck et al., 2020, White et al., 2021). Given the association between risk perceptions of COVID-19 illness severity and greater quitting readiness and attempts, and prior research assessing the messaging effectiveness of linking smoking and COVID-19 (Grummon et al., 2020), smoking cessation messaging that stresses the link between smoking and COVID-19 illness severity may be effective.

While it is not surprising that smokers who believe smoking causes greater COVID-19 effects severity are more ready to quit, it is surprising that this perception is linked to increases in the amount of smoking, even as the perception of greater general COVID-19 symptoms severity, if infected, is linked with decreases in smoking. One potential explanation is that the smoking-related risk perception increases psychological distress, which triggers more frequent smoking. However, this association persisted even when psychological distress was statistically controlled. This finding may align with previous research on associations between smokers’ risk perceptions, beliefs about COVID-19, and quit intentions. Several cross-sectional surveys found that smokers’ perceptions about increased risk of COVID-19 illness severity were associated with greater quit intentions. However, these surveys showed that despite increased quit intentions, a majority of respondents had smoked at the same level or even increased smoking since the pandemic (Elling et al., 2020, Klemperer et al., 2020). A large body of research suggests that intentions do not necessarily predict behavior change (the “intention-behavior gap”), which may drive our current results (Faries, 2016). Among smokers who perceive greater illness severity of COVID-19, some might be motivated to quit but feel that now is just not the right time because of pandemic-related factors or otherwise.

Another potential explanation for the increase in smoking among those perceiving greater smoking-related severity of COVID-19 effects lies in smokers’ judgment of the risk of infection and their perceived self-efficacy of preventive measures. Studies have shown that smokers often underestimate their health risks from smoking (McKenna et al., 1993), and the same may apply to risk of contracting COVID-19 (Park et al., 2021). The present results include constructs from the Health Belief Model (Jones et al., 2015), and suggest an important role for both threat severity and likelihood. Smokers may believe they would face high severity of illness if infected with COVID-19 but are optimistic that they can avoid infection, thereby diminishing the chances of experiencing severe illness. Thus, perceptions of how severe their illness would be if they were infected could have more impact on their amount of smoking than the belief that smoking causes more severe illness, as these smokers feel they can protect against becoming infected and avoid that potential severity.

In addition to the link between risk perceptions and smoking and quitting outcomes, we also found an association between higher psychological distress and increases and decreases in the amount of smoking, increases in readiness to quit, and increases in quit attempts. This finding mirrors prior research showing COVID-19-related stress contributed to both positive and negative changes in smoking (Bommelé et al., 2020). Other studies suggest associations between greater COVID-19-related stress, inhibited quitting efforts (Joyce et al., 2021), and increased smoking (Carreras et al., 2021, Patwardhan and Driscoll, 2020). Psychological distress and negative emotions are common triggers for smoking and relapse (Baker et al., 2004). Particularly in high-stress contexts like the COVID-19 pandemic, tobacco cessation interventions that teach adaptive strategies for stress management are needed. For example, mindfulness-based interventions are efficacious for reducing psychological distress (Khoury et al., 2015) and show promise for smoking cessation (Oikonomou et al., 2017). Mindfulness training teaches people to non-judgmentally observe experiences of stress and craving so that they can purposefully respond rather than automatically react by smoking (Brewer et al., 2013). In the context of the pandemic, it might be useful for clinicians to not only emphasize the association between smoking and COVID-19 illness severity (which could prompt greater readiness to quit), but also equip smokers with mindfulness or other techniques to help them manage stress without smoking.

Finally, our study found that more smokers believed smoking can cause greater severity of COVID-19 illness than believed smoking contributes to greater susceptibility of COVID-19 illness. These beliefs are consistent with much of the scientific literature on the link between smoking and COVID-19 outcomes (Berlin et al., 2020, Heydari and Arfaeinia, 2021, Patanavanich and Glantz, 2020, Reddy et al., 2021, Zhang et al., 2021, Zhao et al., 2020), indicating that smokers’ beliefs are at least somewhat aligned with scientific findings. While this is true for smokers overall, our study found some significant differences by smoker age, ethnicity, and education level. Smokers between the ages of 30 and 44 were more likely than older smokers to believe their smoking could cause greater susceptibility to, and severity of, COVID-19 illness. This lines up with past research finding that smokers who never plan to quit tend to be older and more likely to deny that smoking causes illness (Popova, Majeed, et al., 2018). Similarly, smokers with a college degree were more likely to believe that smoking could cause greater susceptibility to COVID-19 and greater severity of COVID-19 illness than those with less education. Indeed, some research suggests that individuals with lower education are less aware of the health risks of smoking (Siahpush et al., 2006). In addition, higher education is correlated with higher health literacy, which can help people to access and understand relevant health information in the context of COVID-19 (Spring, 2020). Regardless of the perceived impact of smoking, older smokers and Hispanic smokers (compared with White, non-Hispanic smokers) reported greater perceived severity of COVID-19 symptoms if they were to be infected. These beliefs align with widely reported findings concerning profiles of who may be at greater risk of severe illness (Pennington et al., 2021).

### 4.1 Limitations

Our study contains several limitations. First, due to the cross-sectional survey design, causality between variables cannot be confirmed. Second, data were all self-reported, with some measures subject to recall bias since biochemical verification of smoking was not possible in this online survey. Third, this analysis examines only current smokers and thus does not consider the perceptions and outcomes of those who successfully quit during the COVID-19 pandemic. Fourth, our survey was conducted over a two-month period at one specific point during the COVID-19 pandemic. Conducting this study at another time since the beginning of the pandemic may have yielded different results based on the current dissemination of scientific knowledge, vaccine availability, and case rates. Fifth, there may be additional contextual and individual factors not explored in our study that are associated with COVID-19 risk perceptions and smoking behaviors. Finally, our study used some measures that were newly created or newly adapted to address the COVID-19 pandemic. As such, the psychometric properties of these measures were not thoroughly tested prior to the survey.

### 4.2 Conclusions

Smokers’ behavioral responses to the COVID-19 pandemic are associated with perceptions of illness severity, both smoking-related and in general, as well as psychological distress, and demographic and situational factors. In particular, perceived smoking-related COVID-19 effects severity was associated with smoking increases, while perceived general COVID-19 symptoms severity was associated with smoking decreases. Readiness to quit was positively associated with both perceived general and smoking-related COVID-19 illness severity and quit attempts were positively associated with perceived general COVID-19 symptoms severity. As our study found no significant association between perceived susceptibility to COVID-19 and smoking and quitting outcomes, it may be beneficial for public health campaigns to emphasize the severity of COVID-19 illness rather than susceptibility. Public health messaging that emphasizes the link between smoking and COVID-19 illness severity, and also suggests supportive resources for quitting smoking may be particularly effective. During the pandemic, interventions that promote healthy stress management among smokers could be particularly useful both for motivating quit attempts and for supporting successful smoking cessation.

**Author Agreement:**

All authors have reviewed and approved the final manuscript. We confirm that this manuscript has not been published nor is under consideration for publication elsewhere.

**Funding source:**

Research reported in this publication was supported by the Robert Wood Johnson Foundation. The content is solely the responsibility of the authors and does not necessarily represent the views of the Robert Wood Johnson Foundation.

#### CRediT authorship contribution statement

**Amy L. Nyman:** Conceptualization, Methodology, Formal analysis, Writing – original draft, Writing – review & editing. **Claire A. Spears:** Conceptualization, Methodology, Writing – review & editing, Funding acquisition. **Victoria Churchill:** Methodology, Writing – review & editing. **Vuong V. Do:** Methodology, Writing – review & editing. **Katherine C. Henderson:** Methodology, Writing – review & editing. **Zachary B. Massey:** Methodology, Writing – review & editing. **Reed M. Reynolds:** Methodology, Writing – review & editing. **Jidong Huang:** Conceptualization, Methodology, Writing – review & editing, Funding acquisition.

## Declaration of Competing Interest

The authors declare that they have no known competing financial interests or personal relationships that could have appeared to influence the work reported in this paper.

## Figures and Tables

**Table:** 

Measure	Coding/Scoring
*Smoking and quitting behavior*	
Changes in amount of cigarette smoking	“How has your cigarette use changed since February 2020 (the start of the COVID-19 pandemic)?” [Increased use, use stayed the same [mode, reference category], decreased use]
Readiness to quit smoking	“Select the number that indicates where you are now in your thinking about quitting smoking.” [No thought of quitting (0) through I am now taking action to quit (10)] Scale collapsed into three categories for analysis: 0–2 vs. 3–7 vs. 8–10.
Quit attempts	“Since February 2020 (the start of the COVID-19 pandemic), have you ever made a serious attempt to quit smoking? That is, have you stopped smoking for at least one day or longer because you were trying to quit?” [Yes, No [mode, reference category]]
*COVID-19 risk perceptions*	
Smoking-related susceptibility to COVID-19	“Based on what you believe, how much do you agree or disagree with the following statements? Smoking cigarettes can cause me to be more likely to get coronavirus.” [strongly disagree/disagree, neither disagree nor agree [mode, reference category], agree/strongly agree]
Smoking-related severity of COVID-19 effects	“Based on what you believe, how much do you agree or disagree with the following statements? Smoking cigarettes can cause me to have more severe effects of coronavirus.” [strongly disagree/disagree, neither disagree nor agree [mode, reference category], agree/strongly agree]
General susceptibility to COVID-19	“How likely do you think you are to be infected by the coronavirus over the next year?” [Unlikely (1), possible (2), likely (3), almost certain (4), certain (5)]
General severity of COVID-19 symptoms	“On a scale from 0 to 10, how severe do you think your symptoms will be if you become infected with coronavirus?” [I would likely have no symptoms (0) to I would likely die from it (10)]
*Participant characteristics and other covariates*	
SexGender	Male, Female
Age	18–29, 30–44, 45–59, 60+
Race/ethnicity	White non-Hispanic, Black non-Hispanic, Other non-Hispanic, Hispanic
Education	Less than High school, High school graduate, Some college, Bachelor’s degree or higher
Household income	< $30,000, $30,000 - $99,999, ≥ $100,000
ENDS use status	Current user, Recent quitter (quit since Feb 2020), Never use or quit before Feb 2020
Psychological distress	“During the past 30 days, about how often did you feel: 1. Nervous 2. Hopeless 3. Restless or fidgety 4. So depressed that nothing could cheer you up 5. That everything was an effort 6. Worthless” [All of the time (4), Most of the time (3), Some of the time (2), A little of the time (1), None of the time (0)] Responses were summed for a range of 0 to 24.
Self-efficacy for quitting smoking	“If you decided you wanted to quit smoking and never start again, how easy or hard do you think it would be for you to do?” [Very easy (1), Hard but you could do it if you tried (2), Very difficult and you might not be able to do it (3), Almost impossible (4)]
Nicotine dependence	“Please rate how often each of the following statements applies to you. 1. I find myself reaching for cigarettes without thinking about it. 2. I drop everything to go out and buy cigarettes. 3. When I haven’t been able to smoke for a few hours, the craving gets intolerable. 4. I smoke more before going into a situation where smoking is not allowed.” [Never (1), Rarely (2), Sometimes (3), Often (4), Always (5)] Responses were summed for a range of 4 to 20.
Cigarettes harder to obtain	“Since February 2020, cigarettes have been… [Easier to obtain (1), Neither easier nor more difficult to obtain (2), More difficult to obtain (3)]”
Cigarettes less affordable	“Since February 2020, cigarettes have been… [More affordable (1), Neither more nor less affordable (2), Less affordable (3)]”
Time spent in places where smoking is allowed	“Since February 2020, what has changed about the amount of time you spend in places or situations where cigarette smoking is allowed?” [I spend **more time** in places or situations where cigarette smoking is allowed (1), No change (2), I spend **less time** in places or situations where cigarette smoking is allowed (3)]

**Table:** 

	Unweighted n	Weighted % (95% CI)
**Gender**: Male Female	678 545	53.4 (49.9, 56.9) 46.6 (43.1, 50.2)
**Age**: 18–29 30–44 45–59 60+	73 293 390 467	16.6 (13.3, 20.5) 30.5 (27.3, 33.9) 29.7 (26.8, 32.7) 23.3 (20.9, 25.8)
**Race/ethnicity**: White, Non-Hispanic Black, Non-Hispanic Other, Non-Hispanic Hispanic	920 138 65 100	69.4 (65.9, 72.7) 13.3 (11.0, 16.0) 6.6 (4.8, 9.0) 10.7 (8.5, 13.2)
**Education**: Less than High School High School Some College Bachelor’s degree or higher	111 424 476 212	18.1 (15.1, 21.5) 37.4 (34.0, 40.9) 33.2 (30.1, 36.4) 11.4 (9.5, 13.5)
**Household Income**: < $30,000 $30,000 - $99,999 ≥ $100,000	374 619 230	29.7 (26.6, 33.0) 47.1 (43.7, 50.7) 23.2 (20.1, 26.5)
**ENDS use status**: Current user Recent quitter (quit since Feb 2020) Never user or quit before Feb 2020	143 32 1048	15.2 (12.5, 18.3) 2.7 (1.9, 4.0) 82.1 (78.9, 84.9)
**Changes in cigarette smoking** Increased Stayed the same Decreased	189 839 184	16.1 (13.7, 19.0) 68.9 (65.6, 72.1) 14.9 (12.6, 17.6)
**Readiness to quit smoking** Precontemplation (0–2) Contemplation (3–7) Preparation (8–10)	328 741 153	26.4 (23.4, 29.6) 61.5 (58.0, 64.9) 12.1 (10.0, 14.6)
**Quit attempts** No Yes	963 255	79.4 (76.4, 82.1) 20.6 (17.9, 23.6)

## Appendix A Study measures

Appendix B Sample Characteristics and Smoking Outcomes (n = 1,223 current smokers)
